# Supplementary material for: Cellulose Isolation from Tomato Pomace: Part II—Integrating High-Pressure Homogenization in a Cascade Hydrolysis Process for the Recovery of Nanostructured Cellulose and Bioactive Molecules
Source: Foods. 2023 Aug 27;12(17):3221. doi: 10.3390/foods12173221 (PMC10487015; doi:10.3390/foods12173221)
Supplement: Supplementary file 1 [file foods-12-03221-s001.zip › foods-2566511-supplementary.pdf]

Article

Cellulose Isolation from Tomato Pomace: Part II—Integrating High-Pressure Homogenization in a Cascade Hydrolysis Process for the Recovery of Nanostructured Cellulose and Bioactive Molecules

Annachiara Pirozzi [1], Federico Olivieri [2], Rachele Castaldo [2], Gennaro Gentile [2] and Francesco Donsì [1,*]

[1] Department of Industrial Engineering, University of Salerno, Via Giovanni Paolo II, 132, 84084 Fisciano, Italy; apirozzi@unisa.it

[2] Institute for Polymers Composites and Biomaterials, National research Council of Italy, IPCB CNR, Via Campi Flegrei, 34, 80078 Pozzuoli, Italy; federico.olivieri@ipcb.cnr.it (F.O.); rachele.castaldo@cnr.it (R.C.); gennaro.gentile@cnr.it (G.G.)

* Correspondence: fdonsi@unisa.it; Tel.: +39-089-964135

The cellulose fibers obtained from tomato pomace (TP) exhibited distinctive characteristics, appearing as agglomerates with an irregular morphology that retained the original cell structure. In contrast, cellulose fibers isolated from TP using HPH-assisted chemical hydrolysis at different stages of the cascade hydrolysis process, displayed notable differences. These cellulose pulps feature smaller agglomerates of irregular shape, including long needle-like debris with length from 600 to 950 µm and 10 to 30 µm in width. The observed variations in size and shape between TP_Cellulose and the others cellulose pulps can be attributed to the fluid-mechanical stresses exerted during the HPH treatment, which enhanced cellulose defibrillation and resulted in the reduction of fiber length.

SEM analysis (Figure S1.e-h) provided further confirmation of the optical microscopy observations. TP cellulose exhibited a thickness of approximately 20 µm and was organized into individual sheets corresponding to the peel cell layers. In contrast, cellulose obtained from NaOH-HPH, H_2SO_4-HPH, and HPH-TP exhibited a higher degree of defibrillation, with individual fibers separating from fiber bundles. Notably, HPH-TP cellulose displayed fragmented sheets resembling small pieces with an average size of about 100 µm, with a honeycomb-like structure characterized by large cavities and high void fraction. This unique structure resulted in a significantly larger specific surface area compared to TP cellulose, thereby enhancing the techno-functional properties of cellulose, a phenomenon often referred to as fiber activation [1–3]. One practical manifestation of this enhanced functionality was the remarkable capability of HPH-activated TP fibers to act as stabilizers in Pickering emulsions [4].

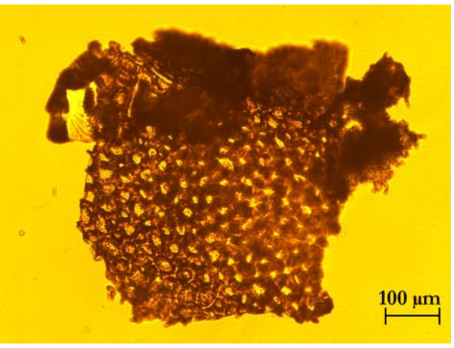

(**a**)

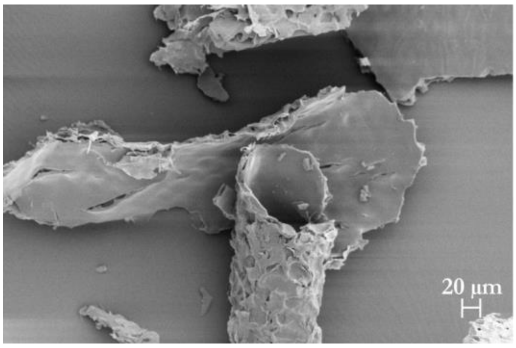

(**e**)

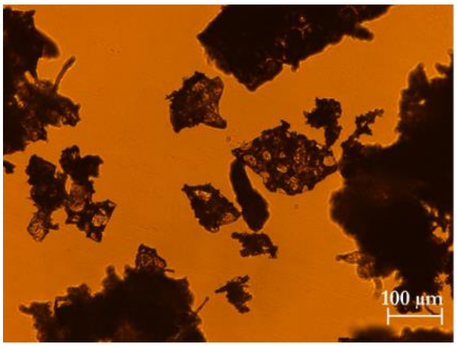

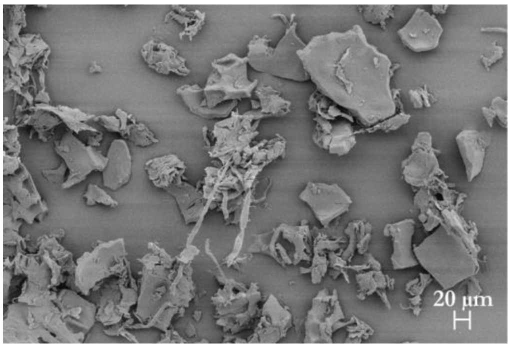

(**b**) (**f**)

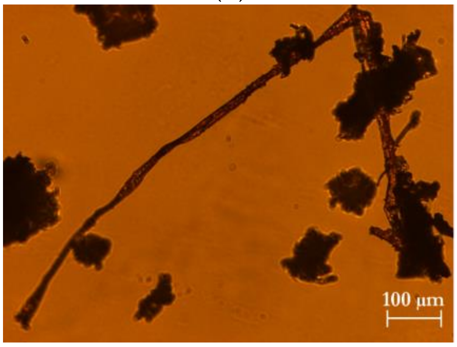

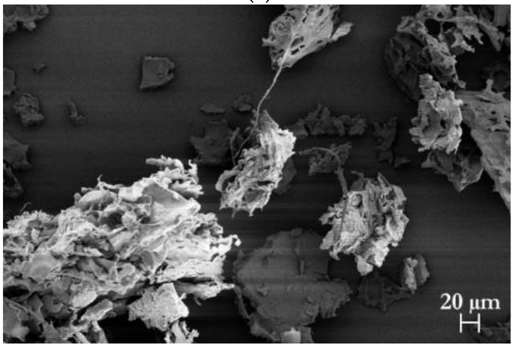

(**c**) (**g**)

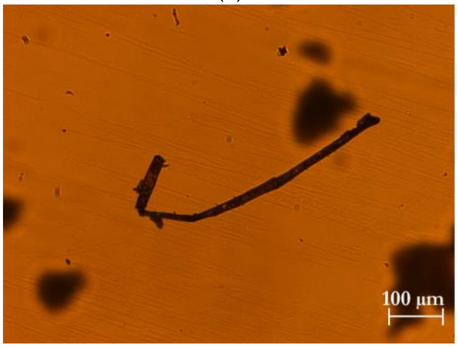

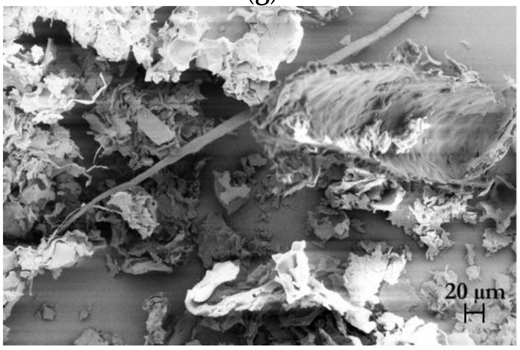

(**d**) (**h**)

Figure S1. (a-d) Optical microscopy at 10× magnification and (e-h) SEM images at 500× magnification of (a, e) TP_Cellulose; (b, f) NaOH-HPH_Cellulose; (c, g) H_2SO_4-HPH_Cellulose; and (d, h) HPH-TP_Cellulose.

References

1. Bi, C.H.; Yan, Z.M.; Wang, P.L.; Alkhatib, A.; Zhu, J.Y.; Zou, H.C.; Sun, D.Y.; Zhu, X. Di; Gao, F.; Shi, W.T.; et al. Effect of high pressure homogenization treatment on the rheological properties of citrus peel fiber/corn oil emulsion. *J. Sci. Food Agric.* **2020**, *100*, 3658–3665.
2. Hua, X.; Xu, S.; Wang, M.; Chen, Y.; Yang, H.; Yang, R. Effects of high-speed homogenization and high-pressure homogenization on structure of tomato residue fibers. *Food Chem.* **2017**, *232*, 443–449.
3. Zhu, X.; Lundberg, B.; Cheng, Y.; Shan, L.; Xing, J.; Peng, P.; Chen, P.; Huang, X.; Li, D.; Ruan, R. Effect of high-pressure homogenization on the flow properties of citrus peel fibers. *J. Food Process Eng.* **2018**, *41*, e12659.
4. Pirozzi, A.; Capuano, R.; Avolio, R.; Gentile, G.; Ferrari, G.; Donsì, F. O/W pickering emulsions stabilized with cellulose nanofibrils produced through different mechanical treatments. *Foods* **2021**, *10*, 1–18.

Disclaimer/Publisher's Note: The statements, opinions and data contained in all publications are solely those of the individual author(s) and contributor(s) and not of MDPI and/or the editor(s). MDPI and/or the editor(s) disclaim responsibility for any injury to people or property resulting from any ideas, methods, instructions or products referred to in the content.
